# Regulation of T cell tissue residency and activation in human PCLS

**DOI:** 10.1186/s12931-025-03397-1

**Published:** 2025-11-15

**Authors:** Tonia Bargmann, Charline Sommer, Lena Stowasser, Sophie Jacob, Louisa Jürgen, Sebastian Konzok, Christopher Werlein, Patrick Zardo, Lavinia Neubert, Danny Jonigk, Hans-Gerd Fieguth, Franziska Dahlmann, Katherina Sewald, Susann Dehmel, Armin Braun

**Affiliations:** 1 Fraunhofer Institute for Toxicology and Experimental Medicine, Preclinical Pharmacology and Toxicology, Nikolai-Fuchs-Str. 1, Hannover, 30625 Germany; 2 Member of the Fraunhofer Cluster of Excellence Immune-Mediated Diseases CIMD, Hannover, Germany; 3 German Center for Lung Research (DZL), BREATH Hanover, Hanover, Germany; 4 Hannover Medical School, Institute of Pathology, Hannover, Germany; 5 Department of Cardiac, Thoracic, Transplantation and Vascular Surgery, Hannover Medical School, Hannover, Germany; 6 RWTH Aachen University Medical Faculty, Institute of Pathology, Aachen, Germany; 7 Klinikum Siloah and Klinikum, Nordstadt Pathology, Hannover, Germany; 8 Hannover Medical School, Institute of Immunology, Hannover, Germany

**Keywords:** PCLS, Ex vivo, Resident memory t cells, Recall response, MHC-I peptides, Polyclonal t cell activation

## Abstract

**Background:**

Resident immune cells are central in shaping the lung’s tissue-specific immunity. Precision-cut lung slices (PCLS) preserve the native tissue microenvironment and are therefore an excellent ex vivo model to analyze residency and functionality of resident memory T cells.

**Methods:**

To study the modulation of tissue residency markers and T cell activation in the native lung niche, we treated PCLS with broad and T cell-specific stimuli and analyzed responses using flow cytometry and mediator secretion analysis. Using TGFβ, anti-CD3/CD28, IL-2 and a pool of MHC-I restricted peptides we analyzed cytokine secretion, CD4^+^/CD8^+^ T cell ratios, and the expression of activation and residency markers.

**Results:**

First, we characterized lung immune cell in PCLS which also revealed that resident memory T cells are abundant in PCLS. We showed that regulation of the tissue residency marker CD103 is dependent on TGFβ or IL-2 signaling in combination with T cell receptor engagement. Further, polyclonal activation of T cells in the tissue reduced tissue secretion of anti-inflammatory cytokines like TGFβ, while increasing the secretion of T cell-associated cytokines like IFNγ, IL-2, and Granzyme B. This shift was supported by an upregulation of T cell activation markers such as CD39, CD137, and Ki-67. Finally, treatment of PCLS with a pool of MHC-I-restricted peptides led to increased secretion of multiple inflammatory effector cytokines associated and a specific activation of tissue resident T cells.

**Conclusion:**

Taken together, we have demonstrated that PCLS provide an excellent platform to modulate tissue resident T cell responses influenced by human lung tissue microenvironment.

**Supplementary Information:**

The online version contains supplementary material available at 10.1186/s12931-025-03397-1.

## Introduction

The lung’s tissue-specific immunity is largely shaped by its resident immune cells, among which, tissue-resident T cells play a significant role in the defense against pathogens [1, 2]. Studying T cell responses in the context of the tissue microenvironment is vital, because differentiation, tissue residency and function are shaped by their specialized environments. In vivo mouse models have been a keystone in dissecting T cell biology. However, their controlled genetics, standardized environments, and uniform disease courses limit their ability to capture the complexity of human immune responses [3]. In contrast, human-derived samples reflect the diversity of tissue microenvironments shaped be individual genetics, pathogen exposure history, and comorbidities. These factors profoundly shape T cell immunity in the lung. Resident memory T cells (T_RMs_) are an abundant T cell population in the non-inflamed human lung, with about 1 × 10^10^ T cells per lung based on histological extrapolations [4]. T_RMs_ arise from antigen-inexperienced T cells, which, upon sustained antigen exposure within peripheral tissues, undergo differentiation into antigen-experienced T_RMs_. The differentiation process is accompanied by an upregulation of residency markers like CD103 and CD49a, as well as markers that indicate antigen encounter like CD69 [1, 5, 6]. This process is facilitated by tissue-derived cytokines and growth factors, including TGFβ, IL-2, IL-15, and IL-33 [1, 7]. Thus, T_RM_ tissue residency is tightly regulated by the local tissue microenvironment via residency marker ligands and tissue derived cytokines [2]. A recall response is when T_RMs_ rapidly respond to previously encountered antigen by secreting inflammatory cytokines and cytotoxic molecules. This makes them a key defense mechanism against respiratory pathogens [8, 9]. Although T_RM_s are abundant in the lung, they do not respond until they are re-exposed to antigen [4]. This is due to their antigen-specificity and tightly regulated mechanisms of inhibitory receptor expression [4, 10].

Recall responses have been studied using isolated human immune cells and in vivo mouse models [11–13]. These models however lack the context of native human lung tissue. Precision-cut lung slices (PCLS) contain tissue-resident immune cells and preserve the native human lung microenvironment. Therefore, they are excellent model to enable physiologically relevant assessments of T cell residency and activation. PCLS are being increasingly used to study innate immunity and response to infections [14–17]. Despite the growing use of PCLS in immunological research, detailed characterization of T cell phenotypes and functional responses within this model remains understudied, hindering our ability to fully interpret and harness its potential.

Presently, we aim to study T cell responses within the human lung issue microenvironment by elucidating the effects of tissue-derived factors such as TGFβ and IL-2 on T cell activation and tissue residency in PCLS. In this study, we provide data on T cell activation towards various stimuli in PCLS, showing that the T cells can be activated in both polyclonal and antigen-specific manners, and tissue residency can be modulated in this valuable model system. Our study provides further support for using PCLS to study tissue-specific immune responses.

## Methods

### PCLS generation and treatment

PCLS generation was conducted as described previously [17, 18]. All PCLS originated from the non-tumor regions of lung tumor patients (Table 1). In short, lung tumor resections were filled with 4% low melting agarose (Fisher Scientific, 10583355) mixed 1:1 with DMEM/F12 (Thermo fisher, 11039021). The filled lung was left to polymerize on ice for 45 min. The lung was cut into slabs, and which were identified as non-tumor bearing by a certified pathologist. The regions of interest were punched out as 8 mm cores and cut into 300–400 μm thick slices using a Krumdiek Tissue slicer (Alabama). After tissue slice generation, they were pooled into petri dishes and washed with DMEM F/12 + P/S (Thermo fisher, 11039021 and 15070063) three times, left over night to rest and were treated the next morning. Two slices were cultivated in 500 µL DMEM/F12 + P/S (medium control). Treatment with anti-CD3/CD28 Dynabeads (Thermo Fisher, 11131D) was conducted by adding 5 µL of beads. IL-2 (Proleukin^®^ S, aldesleukin, Clinigen) was added to reach a final concentration of 1000 U/mL. Carrier freeTGFβ (R&D Systems) was added to a final concentration of 5 ng/mL. To determine the best dosage for recall responses, PCLS were treated with PepTivator^®^ CEF MHC Class I Plus CEF (Miltenyi Biotec, premium grade nmol/peptide) at 2 µL/2 PCLS, 4 µL/2 PCLS or 8µL/2 PCLS. All experiments shown, aside from the representative flow cytometry plots in Fig. 4A were conducted with 8µL/2 PCLS. The respective PepTivator^®^ Negative Control was used at 8 µL/2 PCLS to confirm the reaction specificity. The peptides PepTivator^®^ Influenza A (H1N1) HA, PepTivator^®^ Influenza A (H1N1) NA, PepTivator^®^ SARS-CoV-2 Prot_S (Spike Protein) and PepTivator^®^ SARS-CoV-2 MHC-I (Wild Type Mix) were likewise used at a concentration of 8µL/2 PCLS (Miltenyi Biotec). All samples were treated as technical triplicates. Slices were cultivated for five days after the overnight rest period.

**Table 1:** Donors sex, age, diagnosis and treatment of non-tumor ex vivo tissue derived PCLS

Donor	Sex, Age	Diagnosis	*Ex vivo* Treatment
1	F, 58	NSCLC	TGFβ, anti-CD3/CD28, IL-2
2	F, 68	NSCLC	TGFβ, anti-CD3/CD28, IL-2
3	F, 64	NSCLC	TGFβ, anti-CD3/CD28, IL-2
4	F, 65	Lung Cancer	TGFβ, anti-CD3/CD28, IL-2
5	F, 66	Squamous Cell Carcinoma	Peptides
6	F, 62	NSCLC	Peptides
7	M, 78	Lung Cancer	Peptides
8	M, 79	Lung Cancer	Peptides
9	F, 75	Lung Cancer	Peptides
10	M, 64	Lung Cancer	Peptides

### Dissociation of PCLS

For flow cytometric analysis, six lung slices were pooled, minced into small pieces using surgical scissors, and covered with 1 mL dissociation mix comprised of 1 mg/mL Collagenase D (Merck, 11088858001) and 0.055 mg/mL DNase I (Merck, 4536282001) in DMEM F12. The samples were then left to shake at 200 rpm at 37 °C for 1 h. From this step, samples were constantly kept at 4 °C. After enzymatic dissociation, the slices were mechanically dissociated using a pipette, vortexed for 10 s and filtered through a 100 μm strainer into a 50 mL falcon. The single cell suspension was centrifuged at 350 rcf for 10 min at 4 °C and further processed for conventional flow cytometry of CyTOF measurements.

### Conventional flow cytometry

The pellet was resuspended in 300 µL FACS buffer (PBS + 1% FCS + 2 µM EDTA) containing 1:100 Tru-Stain FcX (Biolegend, 422302) and blocked for 10 min on ice. Subsequently, the suspension was split into three equal parts to stain separate panels. For general immunophenotyping, panel 1 was used: CD45-PerCP, CD3-AF700, CD8-BV510, CD4-BV650, CD56-PE-Cy7, HLA-DR-FITC, CD39-BV421, CD19-PE, and Zombie NIR to distinguish dead cells. For lymphocyte subsets panel 2 was used: CD45-PerCP, CD3-FITC, CD4-BV650, FoxP3-AF647, CD56-APC-Cy7, CD25-BV421, TCRγδ-BV605, CD132-PE, CD122-PE-Cy7, and eFlour 506 as viability marker. And for analyzing activation and memory T cell subsets panel 3 was used: CD45-PerCP, CD3-AF700, CD8-BV510, CD69-BV650, Ki-67-AF647, CD39-BV421, CD103-PE, CD137-PE-Cy7, CD107a-FITC and Zombie NIR to distinguish dead cells (Table 2). Surface stain markers were stained for 30 min at 4 °C. Intracellular staining was conducted for 40 min at 4 °C after 20 min of cell fixation and permeabilization using the FoxP3 Fix Perm Kit according to the manufacturer’s instructions (Thermo fisher, 00552300). Samples were measured using the CytoFLEX S V4-B2-Y4-R0 Flow Cytometer with 10 Detectors and 3 Lasers (Beckman Coulter B96620). Sample analysis was conducted using the FlowJo software (v.10.10.0).

**Table 2:** Antibodies used for flow cytometry including dilution, clone and supplier

Epitope	Fluorophore	Dilution	Clone	Supplier and catalog #
CD45	PerCP	1:70	HI30	Biolegend 304026
CD3	AF700	1:70	UCHT1	Biolegend 300424
CD8	BV510	1:70	RPA-T8	Biolegend 301048
CD4	BV650	1:100	RPA-T4	Biolegend 300536
CD56	PE-Cy7	1:100	HCD56	Biolegend 318318
HLA-DR	FITC	1:70	LN3	Biolegend 327005
CD39	BV421	1:100	A1	Biolegend 328214
CD19	PE	1:100	HIB19	Biolegend 302208
CD103	PE	1:100	Ber-ACT8	Biolegend 350206
CD3	FITC	1:70	UCHT1	Biolegend 300406
FOXP3	AF647	1:100	206D	Biolegend 320114
CD56	APC-Cy7	1:100	HCD56	Biolegend 318332
CD25	BV421	1:100	BC96	Biolegend 302630
TCRγδ	BV605	1:100	11F2	BD 745202
CD122	Pe-Cy7	1:100	TU27	Biolegend 339014
CD132	PE	1:100	TUGh4	Biolegend 338606
CD69	BV650	1:50	FN50	Biolegend 310934
Ki67	APC	1:50	Ki-67	Biolegend 350514
CD137	PE-Cy7	1:100	4B4-1	Biolegend 309817
CD107a	FITC	1:100	H4A3	Biolegend 328606

### CyTOF

The dissociated PCLS samples were stained with the Maxpar^®^ Direct™ Immune Profiling Assay™ kit according to manufacturer’s instructions (Standard Bio Tools). The panel markers included CD45, CD3, CD4, CD8, CD11c, CD14, CD16, CD19, CD20, CD25, CD27, CD28, CD38, CD45RA, CD45RO, CD56, CD57, CD66b, CD123, CD127, CD161, CD294, CCR4, CCR6, CCR7, CXCR3, CXCR5, HLA-DR, IgD, TCRγδ. Samples were measured at the flow cytometry core facility at the Leiden University Medical Center, Netherlands using the CyTOF^®^ XT system, Helios™ system. Analysis and gating strategy were conducted using the FlowJo software (v.10.10.0) (Table 3).

**Table 3:** Gating strategy for cytof analysis of PCLS for each cell type

Cell type	Gating strategy
T cell	CD45^+^/CD3^+^/
CD3 T cell	CD45^+^/CD3^+^/CD4^+^/CD8^−^
CD8 T cell	CD45^+^/CD3^+^/CD4^−^/CD8^+^
Central memory	CD45^+^/CD3^+^/CD4^−^/CD8^+^/CD45RO^+^/CCR7^+^
Effector memory	CD45^+^/CD3^+^/CD4^−^/CD8^+^/CD45RO^+^/CCR7^−^
Naïve	CD45^+^/CD3^+^/CD4^−^/CD8^+^/CD45RO^−^/CCR7^+^
T effector	CD45^+^/CD3^+^/CD4^−^/CD8^+^/CD45RO^−^/CCR7^−^
CD4 T cell	CD45^+^/CD3^+^/CD4^+^/CD8^−^
Th1	CD45^+^/CD3^+^/CD4^+^/CD8^−^/CXCR3^+^/CD294^−^
Th2	CD45^+^/CD3^+^/CD4^+^/CD8^−^/CXCR3^−^/CD294^+^/CCR4^+^
Th17	CD45^+^/CD3^+^/CD4^+^/CD8^−^/CXCR6^+^
Treg	CD45^+^/CD3^+^/CD4^+^/CD8^−^/CD25^+^/CD38^+^
NK Cell	CD45^+^/CD3^−^/CD56^+^
Terminally differentiated NK cells	CD45^+^/CD3^−^/CD56^+^/CD16^−^/CD57^+^
Differentiated cytotoxic NK Cells	CD45^+^/CD3^−^/CD56^+^/CD16^+^/CD57^+^
Cytotoxic NK cells	CD45^+^/CD3^−^/CD56^+^/CD57^−^/CD16^+^
NKT cell	CD45^+^/CD3^+^/CD56^+^
B cells	CD45^+^/CD3^−^/CD19^+^
Mature naïve B cells	CD45^+^/CD3^−^/CD19^+^/IgD^+^/CD20^+^
Late pre-B lymphocytes	CD45^+^/CD3^−^/CD19^+^/CD20^+^
Dendritic Cells	CD45^+^/HLA-DR^+^/CD11c^+^
Classical monocytes	CD45^+^/HLA-DR^+^/CD14^+^
Neutrophils	CD45^+^/CD66b^+^/CD14^+^
γδT cell	CD45^+^/CD3^+^/γδTCR^+^

### Analysis of mediator secretion

Tissue slice supernatants were collected prior to dissociation for flow cytometric analysis. The samples were centrifuged to ensure no cells remained in the supernatant and then stored at − 80 °C before use. Cytokine responses were detected in the supernatant of cultured lung slices using U-Plex MSD (Mesoscale Discovery) panels or Duoplex ELISA (R&D systems). The first MSD panel included: IFNγ, IL-2, TNFα, IL-1β, IL-10, Granzyme A, Granzyme B, IL-18, IL-17 A, IL-21 and Perforin. Additionally, an MSD was used to measure TGFβ1, TGFβ2, and TGFβ3 separately. The multiplex assays were measured using the MESO QuickPlex SE 120MM according to the manufacturer’s instructions. Data was analyzed using the MSD methodical mind Discovery Workbench software (Version 4.0, Mesoscale Discovery). IFNγ and IL-2 were additionally measured using Duoplex ELISA according to manufacturer’s instructions. OD was determined at 450 nm (reference wavelength 540 nm) using the Tecan reader Infinite 200 PRO (Crailsheim, Germany). Cytokine concentrations refer to 2 slices per time point and donor.

### Statistical analysis

Statistical analyses were performed using GraphPad prism 10.1.2 (GraphPad, San Diego, CA). Ratio paired t-tests were performed to compare conditions from the same donor and paired repeated measures (RM) one-way or two-way ANOVAs were used to compare multiple groups as indicated in the figure legends. Data show means and standard deviation (SD). Differences were considered statistically significant at *p* < 0.05 (*), 0.01 (**), 0.001 (***). All data were derived from a minimum of three biological replicates.

## Results

### Immune cell characterization of PCLS

**Fig. 1:**
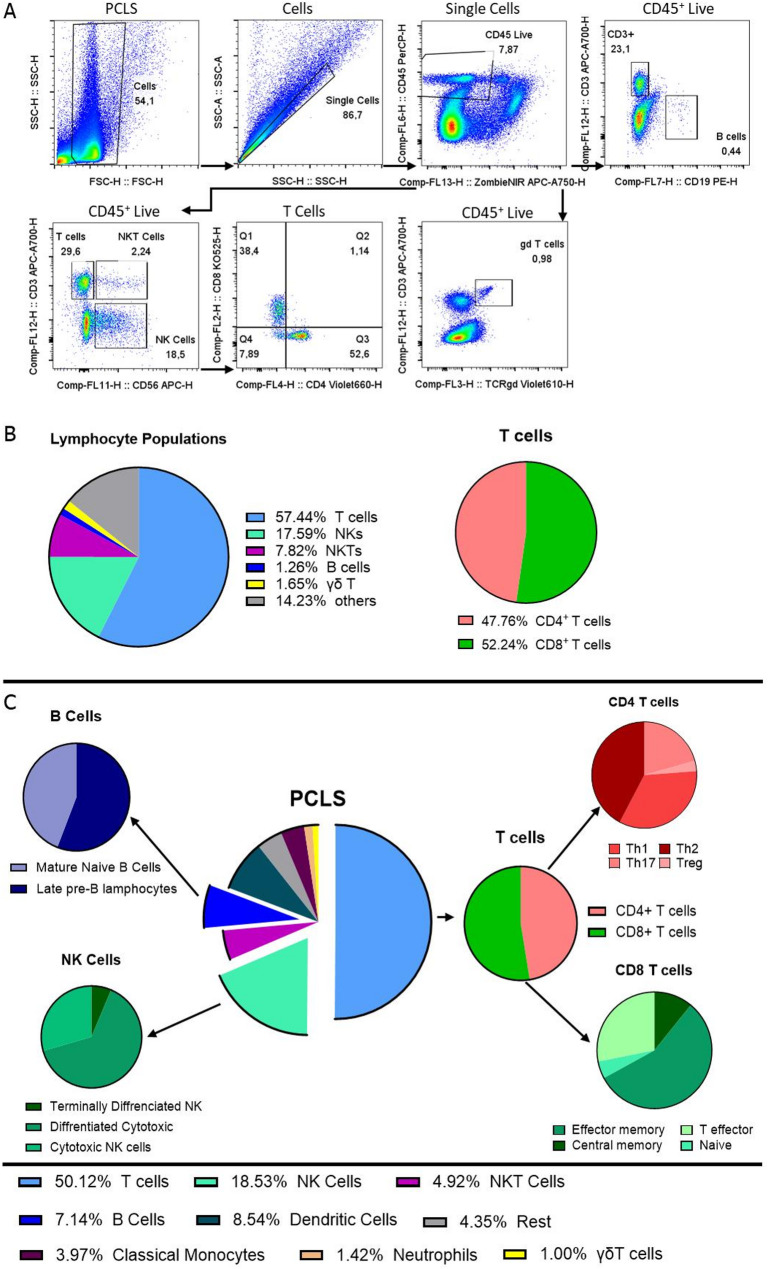
Lymphocyte diversity of the lung is reflected in dissociated PCLS (**A**) Gating strategy of lymphocyte populations via conventional flow cytometric analysis. (**B)** Pie chart displaying the mean frequency of immune cells in human PCLS using conventional flow cytometry, *n* = 5 donors. (**C)** Pie chart representing frequencies of lymphocytes subpopulations in PCLS, *n* = 5 donors. (**D)** Frequencies of immune cell subpopulations in dissociated PCLS as measured by CyTOF *n* = 1 donor

Lung T cell immunity is shaped by its local tissue environment, which is heterogeneous across patients. Thus, we utilized PCLS, to study the regulation of T cell activation and tissue residency in patient-derived native lung tissue. To gain deeper insight into the immune landscape of PCLS and characterize key immune cell subsets, we utilized conventional flow cytometry of dissociated tissue slices. Dissociated PCLS contained 57% CD3^+^ T cells, with the main T cell subsets, CD8^+^ (48% of CD3^+^ T cells) and CD4^+^ T cells (52% of CD3^+^ T cells), as well as 2% γδT cells, 18% NK cells, 8% NKT cells, and 1.2% B cells (Fig. 1A and B). To better mirror the complexity of the immune landscape present in PCLS, we established the CyTOF methodology as a high-content assay to study the immune composition of ex vivo lung tissue. Using a 30-antibody panel via CyTOF analysis, we identified further lymphocyte subsets such as naïve and mature B cells, NK cells in various differentiation states, and subsets of CD8^+^ and CD4^+^ T cell (Fig. 1D). The frequencies of the parent populations of T cells, B cells, NK cells and NKT cells, were comparable to those identified in flow cytometry (Fig. 1B and C ), supporting that composition of lymphocyte populations can be investigated with both conventional and high-content flow cytometry.

### TGFβ and IL-2 in combination with TCR engagement induce tissue residency marker expression on T cells in PCLS

**Fig. 2:**
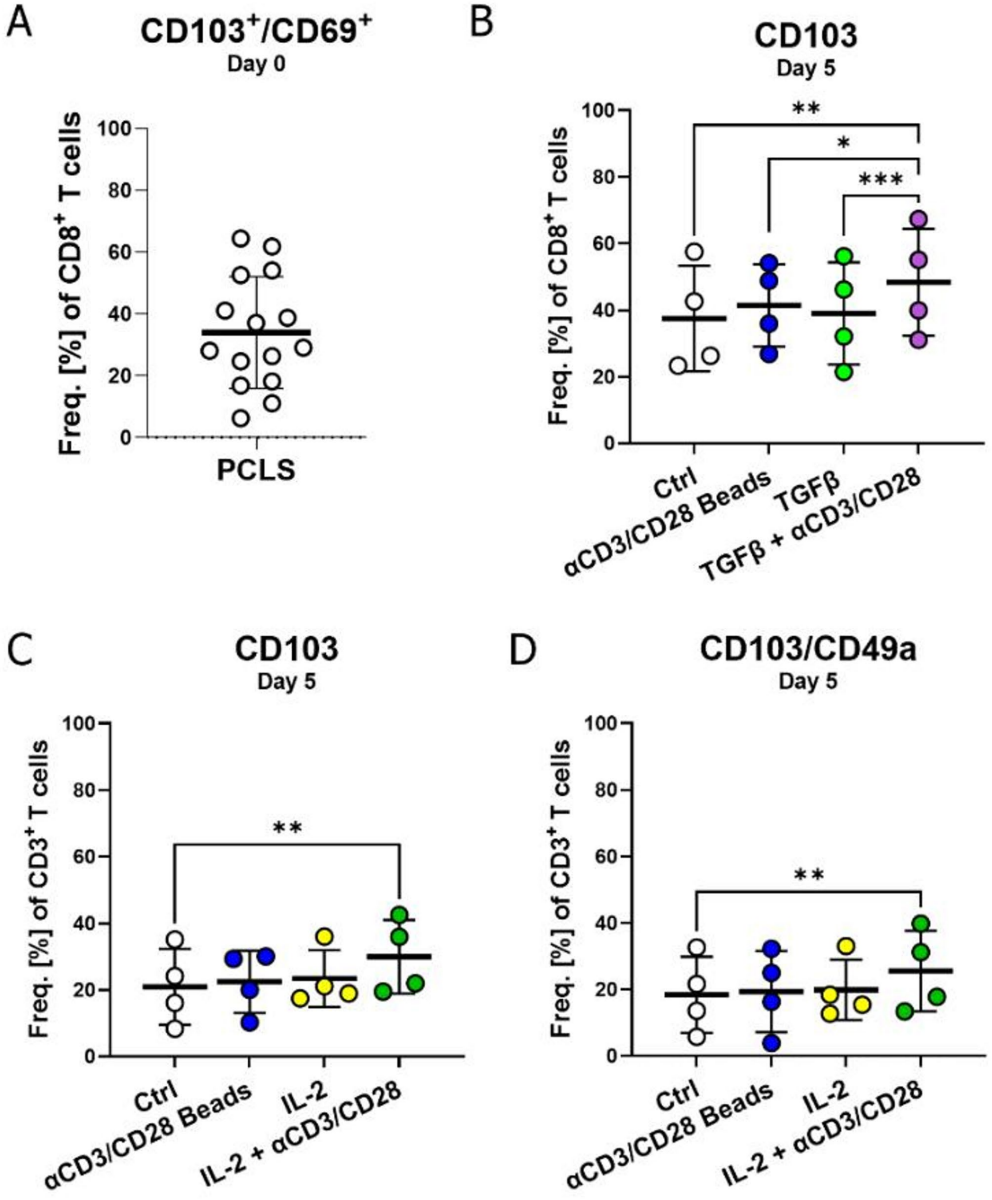
Tissue residency marker CD103 expression on T cells is regulated by TGFβ or Il-2 in combination with T cell receptor engagement. (**A)** Frequency of CD103^+^/CD69^+^ expressing resident memory CD8^+^ cells in PCLS, *n* = 15 donors. (**B)** CD103 expression on CD8^+^ T cells after treatment with anti-CD3/CD28, TGFβ 5 ng/mL or a combination of both for five days. (**C)** Frequency of CD103^+^ (**D**) CD103^+^/CD49a^+^ CD3^+^ T cells after five-day treatment with anti-CD3/CD28, 1000 U/mL IL-2 or a combination of both. *n* = 4 donors, 6 pooled PCLS, four independent experiments measured by flow cytometry. RM paired one-way ANOVA, significance shown are in comparison to medium control. Shown with mean and SD, *p < 0.05, **p < 0.01, ***p < 0.001

A major fraction of T cells in the lung have a resident memory T cell (T_RM_) phenotype and are CD103^+^/CD69^+^/CD8^+^ (mean: 38%, range: 8%−65%) (Fig. 2A). TGFβ signaling plays a central role in the generation of resident memory T cells [19]. Thus, we investigated the effect of TGFβ and TCR stimulation on tissue-resident CD8^+^ T cells in PCLS. This revealed that anti-CD3/CD28 and TGFβ alone are not sufficient to regulate CD103 expression. A combination of anti-CD3/CD28 and TGFβ, however, upregulated the expression of CD103 on CD8^+^ T cells, thereby giving them a residency phenotype (Fig. 2B). Besides TGFβ, IL-2 is also a central cytokine that regulates T_RM_ development and maintenance [20]. Thus, we tested the effects of IL-2 and anti-CD3/CD28 on the expression of the integrins CD103^+^ and CD49a. Indeed, IL-2 in combination with anti-CD3/CD28 stimulation caused an increase in CD3^+^/CD103^+^ T cell frequency as well as CD3^+^/CD103^+^/CD49a^+^ T cell frequency (Fig. 2C and D). In this context, PCLS are an excellent system to study the factors required to induce lung tissue residency.

### Polyclonal T cell activation induces full tissue responses in PCLS

**Fig. 3:**
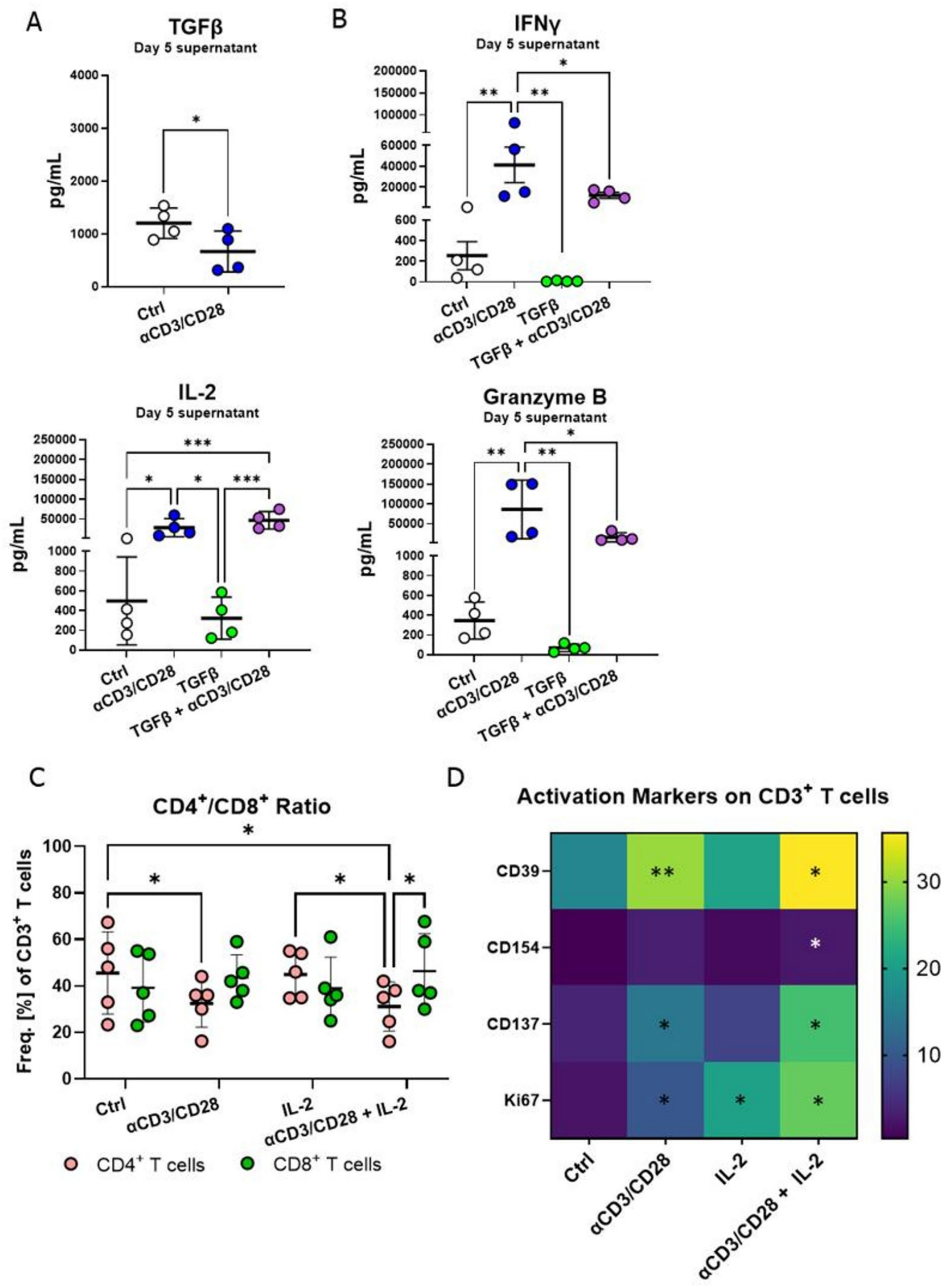
TGFβ, IL-2 and anti-CD3/CD28 differentially modulate T cells effector functions and alter CD4^+^/CD8^+^T cell ratios in PCLS. (**A**) TGFβ in the supernatant of PCLS after five-day treatment with anti-CD3/CD28 and (**B**) IFNγ, IL-2 and Granzyme B secretion by PCLS after treatment with anti-CD3/CD28, TGFβ (5 ng/mL) or a combination of both for five days. *n* = 4 donors, technical triplicates, 2 PCLS/well, four independent experiments. (**C)** Frequency of CD4^+^ and CD8^+^ T cells after five-day treatment with anti-CD3/CD28, 1000 U/mL IL-2 or a combination of both, *n* = 5 donors, 6 pooled PCLS, five independent experiments. (**D**) Heatmap of absolute values representing expression of activation markers CD39, CD154, CD137 and Ki-67 on CD3^+^ T cells after 5-day treatment with anti-CD3/CD28, 1000 U/mL IL-2 or a combination of both, *n* = 6 donors, 6 pooled PCLS, six independent experiments, RM paired one or two-way ANOVA, shown with mean and SD, *p < 0.05, **p < 0.01, ***p < 0.001

To investigate lymphocyte reactivity in the lung slices, we activated CD3^+^ T cells via antigen-independent T cell receptor (TCR) stimulation using anti-CD3/CD28 beads and measured cytokine secretion. This revealed a significant downregulation of the anti-inflammatory cytokine TGFβ in the lung tissue (0.5-fold change) (Fig. 3A). While anti-CD3/CD28 is a potent TCR activator, TGFβ is a strong T cell suppressor, thereby shaping tissue responses. Thus, we studied the effects of single treatment with anti-CD3/CD28, TGFβ alone, and a combination of both. Polyclonal T cell stimulation induced secretion of the effector cytokines IFNγ, IL-2, and Granzyme B, highlighting T cell and tissue functionality. Single TGFβ treatment decreased the baseline secretion of IFNγ and Granzyme B in all measured donors. In combination with anti-CD3/CD28, TGFβ was not sufficient to decrease IFNγ or IL-2 but significantly decreased Granzyme B secretion, showing that the plasticity of immune responses can be represented in PCLS Fig. 3B).

IL-2 signaling and TCR engagement are essential to activate T cells and induce proliferation. To study the effect of global activation of T cells, we treated PCLS with anti-CD3/CD28 and IL-2, alone or in combination. Anti-CD3/CD28 treatment alone, and in combination with IL-2, but not IL-2 alone, resulted in a higher frequency of CD8^+^ T cells in PCLS (Fig. 3C). To confirm T cell activation and proliferation, we measured expression of activation markers like CD39, CD137, CD154, and Ki-67 on CD3^+^ T cells after five days of treatment. A combination of polyclonal T cell activation and IL-2 induced the strongest upregulation of all measured markers and anti-CD3/CD28 alone increased the expression of CD137 and CD39 more so than IL-2 alone (Fig. 3D). These treatments revealed treatment-dependent alterations in lymphocyte composition and activation.

### MHC-I restricted peptides induce tissue-resident T cell activation in PCLS

**Fig. 4:**
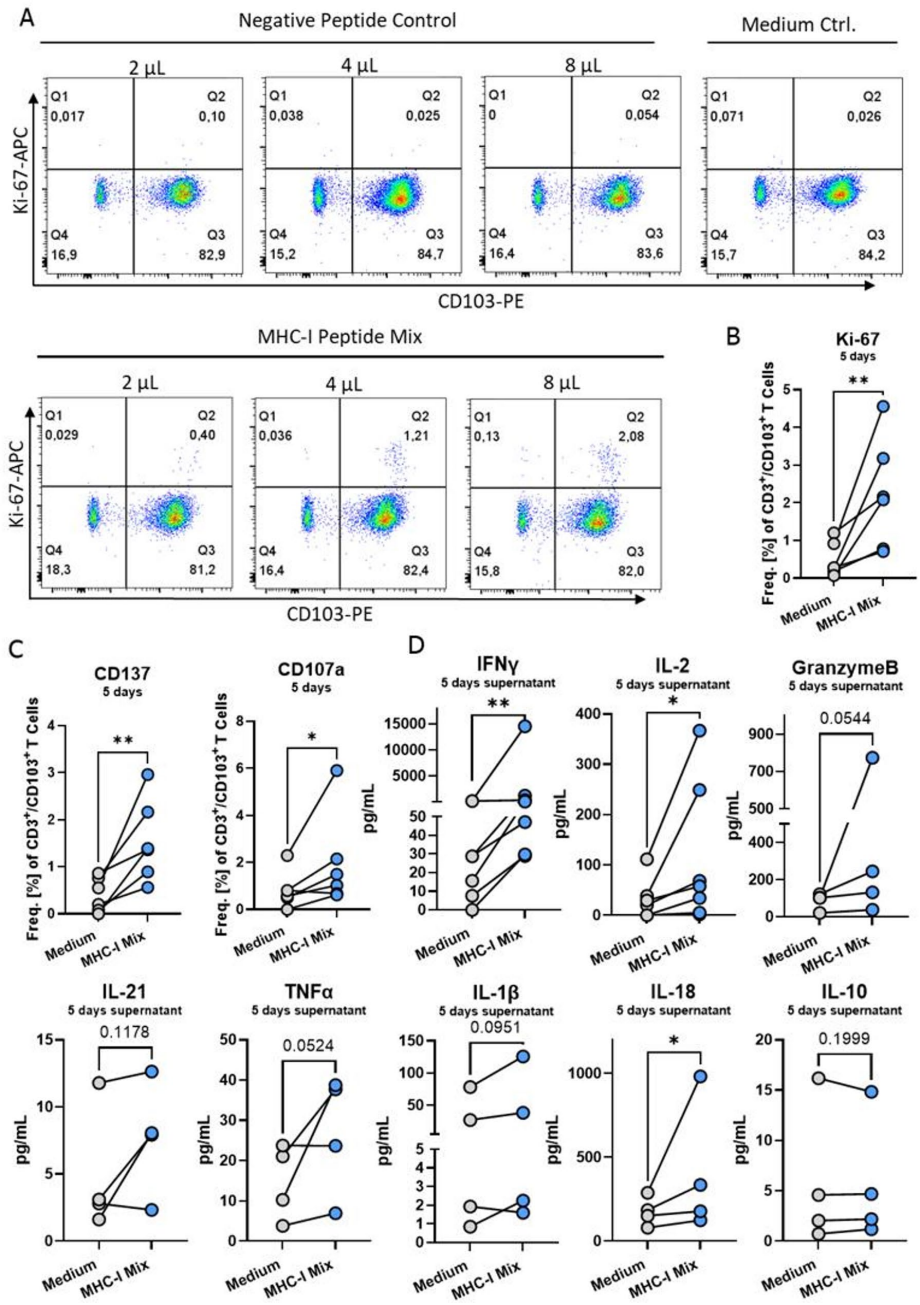
MHC-I peptide mix activates tissue-resident T cells in PCLS. (**A)** Representative flow cytometric plots of Ki-67 and CD103 expression, gated on CD3^+^ T cells treated for five days with medium control, negative control peptides and MHC-I peptide mix with 2 µL/2 PCLS, 4 µL/2 PCLS and 8µL/2 PCLS. (**B)** Ki-67, (**C)** CD137, and CD107a expression on CD3^+^/CD103^+^ T cells in PCLS and (**D**) PCLS supernatant content of IFNγ, IL-2, Granzyme B, IL-21, TNFα, Il-1β, IL-18, and IL-10 with and without 8 µL/2 PCLS MHC-I peptide treatment for five days, *n* = 4 or 6 donors, technical triplicates, 2 PCLS/well for cytokine analysis and 6 pooled PCLS for flow cytometric analysis, four or six independent experiments. Ratio-paired t-test *p < 0.05, **p < 0.01

Next, we aimed to investigate the effects of a more specific stimulus using an MHC-I restricted peptide pool mix. In peripheral blood mononuclear cell (PBMC) assays, peptide mixes are taken up by antigen presenting cells to induce memory T cell activation [21]. We found that, increasing concentrations of the MHC-I peptide mix induced a higher frequency of proliferating resident T cells (CD3^+^/CD103^+^/Ki-67^+^) (Fig. 4A). Proliferation of CD3^+^/CD103^−^ T cells was observed at much lower frequencies (< 1%), indicating primary responsiveness of antigen-experienced T cells (Supplementary Fig. 1). The negative peptide control did not induce T cell proliferation or cytokine secretion (Fig. 4A and B, Supplementary Fig. 2). Furthermore, the MHC-I peptide mix induced expression of the activation marker CD137 on CD3^+^/CD103^+^ T cells of all donors (Fig. 4C). This T cell activation was accompanied by increased tissue secretion of IFNγ and IL-2 (Fig. 1D). CD3^+^/CD103^+^ T cells from five out of six donors also upregulated the degranulation marker CD107a (Fig. 4C). This was complemented by tissue secretion of Granzyme B (Fig. 4D). The secretion of IL-21, TNFα, and IL-1β was donor-dependently regulated, with two donors that responded to stimulation and two that did not. Interestingly, IL-18 secretion was significantly upregulated while IL-10 secretion remained unaffected (Fig. 4D). Finally, while the MHC-I peptide mix induced responses in all donors, specific peptides yielded more donor-specific responses, as evidenced by IFNγ secretion upon stimulation with specific influenza and SARS-COV-2 peptides (Supplementary Fig. 2B).

Taken together, these data demonstrate that tissue-resident T cells in PCLS are specifically activated by MHC-I restricted peptide pools and that this induces involvement of antigen-presenting cells. Further, the local tissue milieu in PCLS influence not only the magnitude of T cell proliferation but also the breadth and strength of cytokine secretion, cytotoxic capacity, and memory phenotypes.

In summary, we demonstrated that T cell tissue residency in PCLS is shaped by TGFβ and IL-2 in combination with TCR engagement. Further MHC-I restricted peptide pools specifically activated resident T cells in native lung tissue. These results highlight that PCLS are an excellent platform to study T cell responses within the human lung tissue microenvironment. In PCLS, the local milieu influences the magnitude of T cell proliferation, cytotoxic capacity, and memory phenotypes.

## Discussion

Presently, our findings highlight how PCLS can be leveraged to unravel the dynamics of human lung-resident T cell activation and function within their native microenvironment. In this study, we characterized the lymphocyte landscape and studied their activation, regulation, and tissue residency in PCLS. PCLS preserve the complexity of the native lung architecture and maintain the interactions of diverse immune subsets, making them a valuable platform for investigating ex vivo immune responses. Here, we demonstrate that PCLS contain immune cells as expected in native lung tissue, retain functional responsiveness to both polyclonal and antigen-specific stimulation, and allow dissection of regulatory roles of cytokines such as TGFβ and IL-2 in shaping tissue-resident T cell phenotypes.

Characterization of immune composition using flow cytometry and CyTOF revealed that PCLS contain a diverse population of T cells, NK cells, NKT cells, B cells, and monocytes. The frequencies of these cells were comparable not only across the two methods but also with regard to current literature on lung immune cells [22, 23], except for monocytes, which showed a lower frequency compared to published histology data [24]. This is likely due to the method of dissociation used, which strongly favors the liberation of lymphocytes and may skew relative frequencies [25, 26]. In comparison to conventional flow cytometry data, CyTOF analysis provided higher resolution by identifying additional subsets such as naïve and mature B cells, NK differentiation stages, and effector/memory T cell subsets with one sample. This is especially advantageous given the fact that PCLS represent a valuable but scarce tissue model, meaning that the number of slices generated, and the number of immune cells isolated from PCLS is limited. Thus, CyTOF is a technique to investigate immune cell populations in-depth with few tissue slices used. Additionally, due to the use of heavy metal isotopes, CyTOF reduces the background and autofluorescence often seen in lung tissue [27]. The concordance between flow cytometry and CyTOF measurements supports the robustness of these methodologies in capturing immune diversity within PCLS. In addition, functional assays confirmed that lymphocytes within PCLS remain responsive after several days of cultivation.

We identified a high proportion of CD8^+^ T_RM_ cells (CD103^+^/CD69^+^) in PCLS, consistent with the lung’s role as a frontline barrier organ. TGFβ plays a significant role in the regulation and maintenance of T_RMs_ [28]. One study revealed that upregulation of the tissue residency marker, CD103, on PBMC T cells requires both TCR stimulation as well as TGFβ [29]. We further confirmed this in our PCLS model within the tissue. IL-2 in combination with anti-CD3/CD28 also promoted tissue residency, evidenced by increasing the expression of CD103^+^/CD49a^+^ on all T cells. These data support existing literature demonstrating that the expression of CD103 on both CD4^+^ and CD8^+^ T cells is regulated by both IL-2 and TGFβ [30, 31]. Our data highlights the role of IL-2, not only in survival but also in regulating the expression of residency markers, emphasizing that a dynamic interplay of activation and cytokine signals orchestrates T_RM_ differentiation.

Polyclonal activation via anti-CD3/CD28 induced robust secretion of IFNγ, IL-2, and Granzyme B by the tissue. In contrast, TGFβ treatment alone suppressed cytokine production, consistent with its well-established immunosuppressive role [32]. Interestingly, combined anti-CD3/CD28 and TGFβ stimulation selectively reduced Granzyme B secretion while maintaining IFNγ and IL-2 levels, suggesting a nuanced regulatory effect that modulates cytotoxicity without completely abrogating effector cytokine responses. These results underscore preserved T cell function that induces tissue cytokine responses in PCLS.

The effect of polyclonal activation using anti-CD3/CD28 on CD4^+^ and CD8^+^ T cell expansion is highly dependent on the model and experimental setup [33, 34]. Thus, understanding this in the context of PCLS is of value. In PCLS, combined IL-2 with anti-CD3/CD28, and anti-CD3/CD28 treatment alone altered the CD4^+^/CD8^+^ T cell balance, favoring CD8^+^ T cells, and induced a significant upregulation of activation and proliferation markers, including CD39, CD137, CD154, and Ki-67. These results indicate that IL-2 amplifies global immune activation in lung tissue, enhancing adaptive responses.

To evaluate antigen specific reactivity of lung resident T cells in this system, we employed an MHC-I restricted peptide pool. Antigen-dependent stimulation induced proliferation and activation of resident T cells, evidenced by Ki-67, CD137, and CD107a expression. Furthermore, secretion of IFNγ, IL-2, and Granzyme B by PCLS was also increased. The secretion of TNFβ, IL-1β and IL-18, but not IL-10, following antigen-specific stimulation indicates a primarily proinflammatory tissue response as a result of T_RM_ activation. Thus, PCLS opens the possibility to study antigen-specific T cell responses in a donor-specific manner. These results confirm that T_RMs_ in PCLS not only retain the capacity for antigen-specific recall responses [35, 36].

## Conclusion

In conclusion, our findings establish PCLS as a physiologically relevant model to study lung immunity, with a specific emphasis on T_RM_ cell responses. This system preserves immune diversity, allows investigation of global and antigen-specific lymphocyte activation, and captures the regulatory roles of cytokines in shaping tissue-resident immunity. The ability of PCLS to recapitulate T_RM_ differentiation and functional responses highlights their value for studying immune regulation and therapeutic strategies aimed at modulating tissue-resident immunity in the lung.

## Supplementary Information

Supplementary Material 1.

## Data Availability

The datasets generated during and/or analyzed during the current study are available from the corresponding author on reasonable request.

## Abbreviations

CD: Cluster of Differentiation
IFNγ: Interferon Gamma
IL: Interleukin
NK: Natural Killer cell
NKT: Natural Kille T cell
PBMC: Peripheral blood mononuclear cells
PCLS: Precision-cut lung slices
MHC-I: Major histocompatibility complex class one
TCR: T cell receptor
TGFβ: Transforming growth factor beta
TNF: Tumor necrosis factor
T_RM_: T resident memory cells
