# Supplementary material for: Regulation of T cell tissue residency and activation in human PCLS
Source: Respir Res. 2025 Nov 15;26:319. doi: 10.1186/s12931-025-03397-1 (PMC12619209; doi:10.1186/s12931-025-03397-1)
Supplement: Supplementary file 1 — Supplementary Material 1. [file 12931_2025_3397_MOESM1_ESM.docx]

# Supplements


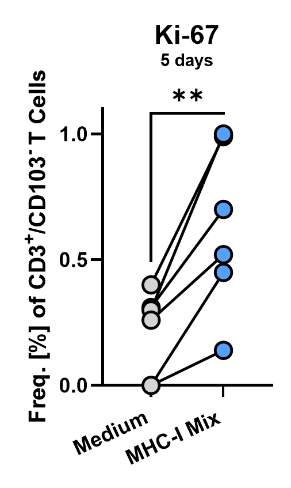


**Supplementary Figure 1. Ki-67 expression on CD3^+^/CD103^-^ T cells after treatment with MHC-I peptide mix.** n=6 donors, technical triplicates, 6 pooled PCLS for flow cytometric analysis, six independent experiments. Ratio-paired t-test, **p<0.01.


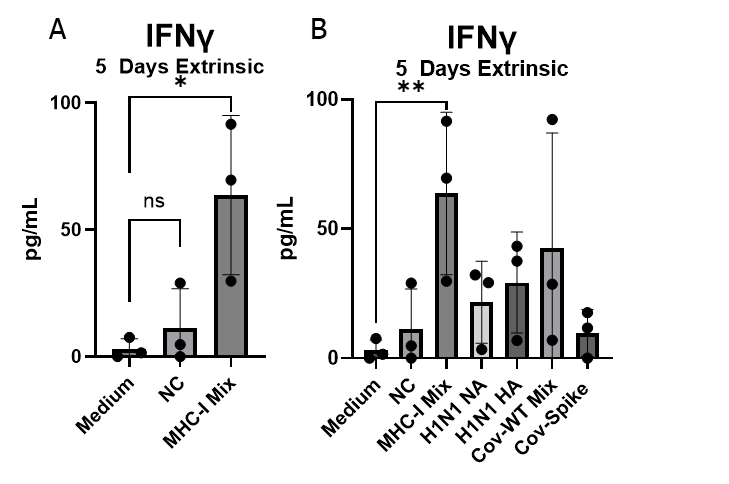


**Supplementary Figure 2. Viral peptide mixes induce IFNγ secretion by PCLS.** A) IFNγ secretion upon PCLS treatment with medium control, negative control and MHC-I peptide mix. B) n=3, donors, technical triplicates, 2 PCLS/well, four independent experiments, shown with mean and SD, paired One-way ANOVA *p<0.05, **p<0.01
